# Reply to Rossi et al

**DOI:** 10.1093/cid/cix627

**Published:** 2017-07-22

**Authors:** Michelle S Hsiang, Nikhil Ranadive, Stanley Chitundu, Nyasatu Ntshalintshali, Bryan Greenhouse

**Affiliations:** 1 Department of Pediatrics, University of Texas Southwestern Medical Center, Dallas; 2 Malaria Elimination Initiative, Global Health Group; 3 Department of Pediatrics, University of California, San Francisco Benioff Children’s Hospital; 4 Global Health Sciences; 5 Department of Medicine, San Francisco General Hospital, University of California; 6 Emory University School of Medicine, Atlanta, Georgia; 7 National Malaria Control Programme; 8 Clinton Health Access Initiative, Mbabane, Swaziland

To the Editor—We thank Rossi and colleagues for sharing their findings from Cambodia [1], which complement our recent article reporting limitations of rapid diagnostic testing in patients with suspected malaria from Swaziland, a low-endemic country in southern Africa aiming to eliminate malaria [2]. Using polymerase chain reaction (PCR) as gold standard, they performed a diagnostic accuracy evaluation of rapid diagnostic testing (RDT) to diagnose *Plasmodium falciparum* in subjects with suspected malaria. Sensitivity was low at 72% (compared to 52% in our study). Low-density infection, defined as <100 parasites/μL, explained 75% of false-negative results (compared to 76% in our study). With the large sample size of 4382 patients, sampling of all RDT negatives (vs selective sampling employed in our study), and use of quantitative PCR, the study is a useful addition to the few published studies on performance of RDT to assess symptomatic malaria in low-transmission settings [1, 3, 4].

As malaria transmission declines, the proportion of low-density infection among symptomatic as well as asymptomatic individuals increases [5–7]. It is generally assumed that symptomatic individuals will present with high-density infection; however, low-density infections accounted for 24% of all PCR-positive cases, compared to 22% in our study (taking into accounting the sampling of RDT negatives). Given the low prevalence of infection in these settings [8, 9], the unexpectedly high proportion of low-density infection cannot solely be explained by background parasitemia. Rather, patients in low-endemic settings may have a lower pyrogenic threshold for malaria due to decreased immunity, other host factors, or virulence of the parasite [10]. Interestingly, *Plasmodium falciparum* strains from Cambodia have been associated with a lower pyrogenic threshold than some African and American strains [10]. Early access to care, before the parasite has undergone multiple cycles of replication, would be facilitated by village malaria workers in the Rossi et al study and may also explain the low parasite densities observed.

Missed low-density infections represent missed opportunities to prevent further transmission. They also represent missed opportunities for transmission reduction activities in the community, as passively identified cases may trigger targeted interventions such as active case detection and vector control. On the flip side, overdiagnosis is also a problem. We would like to note that the false-positivity rates, or the percentage of healthy individuals who incorrectly receive a positive test result, were incorrectly reported in both studies. The correct false-positive rates were low at 5.9% in Swaziland and 0.3% in Cambodia (not 32% and 11%, respectively). However, due to the low prevalence of malaria, positive predictive values (PPVs) were compromised. Rossi et al report a higher PPV than our study (89%, compared to 67% in Swaziland), but a PPV of 89% still equates to overtreatment in roughly one-tenth of patients, and potential “overintervention” in the communities where activities were triggered by passively detected cases. A new RDT with reported sensitivity 10 times higher than current RDTs has recently been launched. While its use has potential to reduce transmission [11], there may be compromises in specificity due to the fact that the target antigen can persist in the bloodstream for several weeks, despite clearance of infection. Confirmatory testing with a highly specific test, as is done with human immunodeficiency virus testing, may be one solution. Certainly, as alternative diagnostic approaches are being considered for malaria, the balance of predictive values, sensitivity, specificity, as well as impact at individual and community levels, will need to be thoughfully considered.

## Notes

***Financial support.*** This work was supported by the Swaziland Ministry of Health; the Bill and Melinda Gates Foundation (grant number A121292); the National Institute of Allergy and Infectious Diseases at the National Institutes of Health (grant number AI101012 to M. S. H.); Burroughs Wellcome Fund/American Society for Tropical Medicine and Hygiene Fellowship (grant number A120079 to M. S. H.); the Horchow Family Fund (grant number 5300375400 to M. S. H.); and the Gilead Translational Fellows Grant (NCE A122737 to N. R.).

***Potential conflicts of interest.*** All authors: No reported conflicts of interest. All authors have submitted the ICMJE Form for Disclosure of Potential Conflicts of Interest. Conflicts that the editors consider relevant to the content of the manuscript have been disclosed.
